# High Blood Pressure in Adolescents of Curitiba: Prevalence and Associated Factors

**DOI:** 10.5935/abc.20160044

**Published:** 2016-05

**Authors:** Rodrigo Bozza, Wagner de Campos, Valter Cordeiro Barbosa Filho, Antonio Stabelini Neto, Michael Pereira da Silva, Renato Silva Barbosa Maziero

**Affiliations:** 1 Universidade Federal do Paraná (UFPR), Curitiba, PR - Brazil; 2 Universidade Federal de Santa Catarina (UFSC), Florianópolis, SC - Brazil; 3 Universidade Estadual do Norte do Paraná (UENP), Jacarezinho, PR - Brazil

**Keywords:** Arterial Blood Pressure, Hypertension/genetics, Waist Circumference, Risk Factors, Adolescent

## Abstract

**Background:**

Arterial hypertension is a major public health problem and has increased considerably in young individuals in past years. Thus, identifying factors associated with this condition is important to guide intervention strategies in this population.

**Objective:**

To determine high blood pressure prevalence and its associated factors in adolescents.

**Methods:**

A random sample of 1,242 students enrolled in public schools of the city of Curitiba (PR) was selected. Self-administered questionnaires provided family history of hypertension, daily energy expenditure, smoking habit, daily fat intake, and socioeconomic status. Waist circumference was measured following standardized procedures, and blood pressure was measured with appropriate cuffs in 2 consecutive days to confirm high blood pressure. Relative frequency and confidence interval (95%CI) indicated high blood pressure prevalence. Bivariate and multivariate analyses assessed the association of risk factors with high blood pressure.

**Results:**

The high blood pressure prevalence was 18.2% (95%CI 15.2-21.6). Individuals whose both parents had hypertension [odds ratio (OR), 2.22; 95%CI 1.28-3.85] and those with high waist circumference (OR, 2.1; 95%CI 1.34-3.28) had higher chances to develop high blood pressure.

**Conclusion:**

Positive family history of hypertension and high waist circumference were associated with high blood pressure in adolescents. These factors are important to guide future interventions in this population.

## Introduction

Arterial hypertension accounts for approximately 54% of cerebrovascular diseases and 47% of ischemic heart diseases worldwide, causing approximately 13% of all deaths per year, being, thus, a serious public health problem.^1,2^

Although the major repercussions of arterial hypertension do not manifest in young individuals, it is suggested that children and adolescents with high blood pressure (prehypertension and arterial hypertension) are more likely to become hypertensive adults.^3^ That is worrying, because the prevalence of high blood pressure in young individuals has increased considerably in past decades.^4,5^

In Brazil, a systematic review has identified 21 studies aimed at identifying high blood pressure in the Brazilian young population; however, a large part of those studies has not used representative population samples, in addition to having failed to classify blood pressure and to use proper assessment criteria.^6^ Those limitations can have important implications in estimating high blood pressure prevalence and in defining the factors associated with it.

Nevertheless, it is important to identify possible factors associated with high blood pressure in Brazilian adolescents, such as family history of arterial hypertension,^7-9^ sex,^10-13^ age,^14^ socioeconomic aspects,^13,15,16^ fat intake,^17,18^ energy expenditure,^10,13,16^ smoking,^10,16^ abdominal obesity,^19,20^ and cardiorespiratory fitness.^21^

The identification of population subgroups at risk for developing high blood pressure can better direct prevention strategies in the Brazilian young population.^9,22^ Thus, the present study aimed at determining the prevalence of high blood pressure and its associated factors in a representative sample of adolescents of the public school system.

## Methods

This cross-sectional study was performed from August 2010 to June 2011 with adolescents enrolled in public middle and high schools of the city of Curitiba, Paraná state.

To estimate sample size, the following parameters were considered: (i) population of 115,524 adolescents; (ii) confidence level of 95%; (iii) sampling error of 2%; (iv) arterial hypertension prevalence of 7.4%,^16^ adding up to a minimum sample of 982 adolescents. A design effect of 1.5 was added to correct the error related to multistage sample selection, as was a 30% margin for possible losses and refusals. Therefore, the total sample was estimated in 1,276 adolescents.

The probabilistic sample was selected by use of multiple stages. In the first stage, all public middle and high schools in the city of Curitiba were listed and stratified according to the nine administrative regions of the city. In the second stage, five schools were randomly selected from each administrative region, and, in the third stage, a simple random selection of one to three classes at each school was performed. The adolescents received an assent term, and their parents, a consent term, explaining the research objectives and procedures, to be completed and signed.

The family history of arterial hypertension was obtained with a questionnaire sent to the adolescent's parents or guardians, which indicated if the biological father and/or mother had arterial hypertension.

The waist circumference was measured at a level midway between the last costal arch and the iliac crest, following standard procedures.^23^ The increased value for each sex and age group was determined according to the proposal by Freedman et al.^24^ as ≥ 90^th^ percentile.

The daily energy expenditure (kcal/kg/day) was obtained from a 3-day activity record developed by Bouchard et al.,^25^ the mean value obtained being considered. The individuals were classified into sample quartiles according to sex and age group.

The cardiorespiratory fitness was assessed by using the shuttle run test proposed by Léger et al.^26^ The individuals were classified into sample quartiles according to sex and age group.

To define smoking, the adolescents completed the *Youth Risk Behavior Survey Questionnaire*, translated and validated by Guedes and Lopes.^27^ That questionnaire inquires how many days, in the preceding 30 days, cigarettes were smoked. The individuals were classified into three categories as follows: cigarettes smoked 10 to 30 days; cigarettes smoked 1 to 9 days; and no cigarette smoked in the preceding 30 days.

The total fat intake was obtained by completing a food frequency questionnaire developed for the Brazilian population by Sichieri and Everhart.^28^ To maintain data quality, the cases with energy expenditure greater than 6,000 kcal or lower than 500 kcal were excluded.^29^ According to the American Heart Association, in a proper food intake, total fat intake should be < 30%.^30^

The socioeconomic status was classified according to the criteria established by the Brazilian Association of Research Companies.^31^ In the present study, the participants were classified into the following four socioeconomic levels: I, lowest socioeconomic level (classes D and E); II, classes C1 and C2; III, classes B1 and B2; and IV, highest socioeconomic level (classes A1 and A2).

Blood pressure was measured by using the auscultatory method, following the parameters established in the fourth report of the *National High Blood Pressure Education Program*.^4^ Before that, arm circumference was measured to enable the choice of the cuff size [child (16 - 22 cm), small adult (23 - 26 cm) and adult (27 - 34 cm)].^4^ In addition, the adolescents were asked about having already had their blood pressure assessed before.

Two blood pressure readings were taken at a 5-minute interval, the mean value being considered in this study. If the readings differed more than 2 mm Hg, the protocol would be repeated.^4^

In accordance with *The Fourth Report on the Diagnosis, Evaluation, and Treatment of High Blood Pressure in Children and Adolescents*,^4^ the cutoff values used, according to sex, age and height percentile, were as follows: arterial hypertension - systolic blood pressure (SBP) and/or diastolic blood pressure (DBP) ≥ 95^th^ percentile; prehypertension - SBP and DBP values < 95^th^ percentile and ≥ 90^th^ percentile. Individuals with blood pressure values > 120/80 mm Hg were considered prehypertensive.^4^

Adolescents classified as prehypertensive or hypertensive were reassessed at a subsequent visit on the day following the first assessment, in which case the second blood pressure measurement was considered for the analysis. At both the first and subsequent visit, blood pressure was measured by only one trained professional to prevent interobserver disagreement.

Continuous data were described by use of measures of central tendency and dispersion for both sexes. Continuous data were compared between sexes by use of independent *t* and Mann-Whitney U tests, based on normality of data.

Categorical variables were described by use of relative frequency distribution and its respective 95% confidence interval (95%CI). The chi-square test for heterogeneity was used to compare the differences in the prevalences of prehypertension, arterial hypertension and high blood pressure (prehypertension and arterial hypertension) between sexes, and to compare the high blood pressure prevalences in the first and second visits.

The association between independent variables and high blood pressure was assessed by use of bivariate and multivariate analysis (logistic regression). The variables with a p value < 0.25 on bivariate analysis were selected for the multivariate model. The magnitude of the association between the independent variables and high blood pressure was expressed as odds ratio (OR) and its respective 95%CI. The prevalence and association analyses were corrected with the complex sample design, using the 'complex sample' command of the Statistical Package for the Social Science (SPSS) software, version 17.0. The significance level adopted for all analyses was p < 0.05.

The methodology of this study was approved by the Research Ethics Committee of the Health Science Sector of the Paraná Federal University (UFPR), and abided by the ethical guidelines established by the 196/96 Resolution of the Brazilian National Health Council (CAAE 04414712.3.0000.0102) on the 25^th^ October, 2012.

## Results

Data of 1,812 adolescents were collected. Of those, 570 adolescents meeting at least one exclusion criterion were excluded from the final analysis as follows: 28 individuals were at least 18 years old; 22 were on anti-hypertensive drugs; 84 female adolescents were on oral contraceptives and one was pregnant; 172 adolescents did not deliver the family history of arterial hypertension of both parents; 239 completed the questionnaires incorrectly; 46 underwent neither anthropometric assessments nor the cardiorespiratory fitness test; and 70 individuals did not undergo blood pressure reassessment.

Therefore, this study's final sample comprised 1,242 adolescents aged between 11 and 17 years, 646 of whom (52.01%) were females. The age group distribution was as follows: 11-12 years, 363 individuals (56.2% females and 43.8% males); 13-15 years, 697 individuals (52.5% females and 47.5% males); and 16-17 years, 182 individuals (41.8% females and 58.2% males). Of the total sample, 34.06% had never undergone blood pressure assessment.

Table 1 shows the sample's continuous variables with their central tendency and dispersion.

**Table 1:** Central tendency and dispersion values of the variables according to sex in adolescents

	**General**	**Female**	**Male**
Age, years*	14.1 (1.6)	14.0 (1.5)	14.3 (1.6)†
Height, m‡	1.60 (1.5-1.7)	1.58 (1.5-1.6)	1.65 (1.6-1.7)†
Body mass, kg‡	52.7 (44.8-60.8)	50.7 (44.0-58.5)	54.7 (45.6-62.9)†
Waist circumference, cm‡	67.3 (62.6-72.5)	65.5 (61.5-71.4)	69.0 (64.7-73.7)†
Daily energy expenditure, kcal/day‡	40.0 (36.6-44.7)	38.7 (36.0-42.3)	41.6 (38.0-46.9)†
VO_2max_, mL.kg^-1^.min^-1^*	42.9 (6.5)	39.3 (4.6)	47.0 (5.8)†
Total fat, g‡	87.0 (54.7-138.2)	80.5 (50.7-128.8)	91.9 (60.1-148.6)†
SBP, mmHg*	109.7 (10.72)	108.4 (9.89)	111.1 (11.4)†
DBP, mmHg*	70.03 (7.69)	70.04 (7.67)	70.02 (7.71)

* Mean and standard deviation;

† differences between the sexes p<0.01;

‡ median and interquartile interval. VO_2max_: maximum oxygen consumption; SBP: systolic blood pressure; DBP: diastolic blood pressure.

When comparing continuous data between sexes, all variables, except for DBP, were significantly higher in male individuals (p < 0.01).

Table 2 shows the prevalences of arterial hypertension family history, smoking, total fat intake and socioeconomic level for both sexes.

**Table 2:** Prevalence (%) and confidence interval (95%CI) of the categorical variables in adolescents according to sex

	**General**	**Female**	**Male**
	**% (95%CI)**	**% (95%CI)**	**% (95%CI)**
**Family history**			
No parent	74.7 (71.1-78.1)	71.1 (67.1-74.9)	78.8 (74.7-82.3)
One parent	22.4 (19.2-25.8)	25.3 (21.8-29.0)	19.1 (15.6 - 23.1)
Both parents	2.9 (2.1-4.1)	3.6 (2.5-5.1)	2.1 (1.2-3.8)
**Smoking, days**			
None	93.1 (91.5-94.4)	93.8 (91.3-95.6)	92.3 (90.2-93.9)
1 to 9	4.6 (3.6-5.8)	4.1 (2.7-6.3)	5.1 (3.9-6.7)
10 to 30	2.3 (1.5-3.7)	2.1 (0.9-4.5)	2.7 (1.9-3.8)
**Total fat intake**			
Adequate	46.7 (42.9-50.5)	45.6 (41.4-49.8)	47.9 (42.6-53.2)
Inadequate	53.3 (49.5-57.1)	54.4 (50.2-58.6)	52.1 (46.8-57.4)
**Socioeconomic level**			
A1-A2	5.6 (4-7.8)	4.8 (2.9-7.6)	6.6 (4.2-10.1)
B1-B2	62 (57.6-66.2)	60.1 (53.4-66.4)	64.1 (59.6-68.4)
C1-C2	31.2 (26.6-36.1)	34 (28.0 -40.7)	27.9 (22.5-34.1)
D-E	1.2 (0.7-2.1)	1.1 (0.5-2.4)	1.4 (0.7-2.7)

Table 3 shows the prevalences of prehypertension and arterial hypertension on the two visits. Regarding the general prevalence of high blood pressure (prehypertension and arterial hypertension), a significant difference (X^2^ = 523.1; p < 0.01) between the first (33.0%; 95%CI 29.1-37.1) and second visit (18.2%; 95%CI 15.2-21.6) was observed.

**Table 3:** Prevalence (%) and confidence interval (95%CI) for prehypertension and arterial hypertension on both assessment visits of adolescents according to sex

	**General**	**Female**	**Male**
**Visit**	**% (95%CI)**	**% (95%CI)**	**% (95%CI)**
**First**			
Normal	67.0 (62.9-70.9)	68.3 (62.6-73.4)	65.6 (61.4-69.7)
Prehypertensive	14.1 (12.6-15.7)	13.4 (11.4-15.6)	15.0 (12.5-17.7)
Hypertensive	18.9 (15.6-22.7)	18.4 (14.1-23.6)	19.4 (16.2-23.1)
**Second**			
Normal	81.8 (78.4-84.8)	81.2 (76.2-85.3)	82.5 (78.6-85.9)
Prehypertensive	6.0 (4.3-8.5)	6.1 (4.0-9.3)	5.9 (3.7-9.3)
Hypertensive	12.2 (8.5-14.6)	12.7 (9.7-16.3)	11.6 (9.3-14.3)

Considering only the values of the second visit, no significant difference between sexes was observed in the prevalences of prehypertension (X^2^ = 0.01; p = 0.91) and arterial hypertension (X^2^ = 2.11; p = 0.15).

At the second visit, the high blood pressure prevalence was 17.5% (95%CI 14.1-21.4) in the male sex, and 18.8% (95%CI 14.7-23.8) in the female sex, showing no significant difference between sexes (X^2^ = 1.17; p = 0.28).

Table 4 shows the bivariate and multivariate associations of the variables analyzed with high blood pressure in adolescents. The multivariate model was adjusted to the variables with p < 0.25 on bivariate analysis [family history of arterial hypertension, waist circumference, maximum oxygen consumption (VO_2max_), total fat intake, and socioeconomic level]. On multivariate model, high blood pressure associated (p < 0.05) with family history of arterial hypertension, waist circumference and total fat intake.

**Table 4:** Factors associated with high blood pressure in adolescents

**Variables**	**Bivariate analysis**			Multivariate analysis*	
	**OR (95%CI)**	**p value**		**OR (95%CI)**	**p value**
**Sex**		0.61			
Female	1				
Male	0.92 (0.65 - 1.30)				
**Age group, years**		0.41			
11-12	1				
13-15	0.72 (0.43 - 1.22)				
16-17	0.68 (0.36 - 1.30)				
**Family history of arterial hypertension**		0.04			0.03
No parent	1			1	
One parent	1.19 (0.86 - 1.65)			1.12 (0.82 - 1.53)	
Both parents	2.11 (1.19 - 3.73)			2.22 (1.28 - 3.85)	
**Waist circumference**		0.002			
Normal	1			1	0.003
Increased	2.19 (1.39 - 3.44)			2.10 (1.34 - 3.28)	
**Daily energy expenditure **		0.4			
Q1 (high)	1				
Q2	1.26 (0.89-1.78)				
Q3	1.15 (0.82-1.61)				
Q4 (low)	0.94 (0.66-1.35)				
**VO**_**2max**_		0.18			0.34
Q1 (high)	1			1	
Q2	1.28 (0.58 - 2.85)			1.27 (0.57 - 2.81)	
Q3	1.75 (0.85 - 3.61)			1.62 (0.80 - 3.30)	
Q4 (low)	1.79 (0.98 - 3.24)			1.61 (0.88 - 2.95)	
**Smoking, days**		0.61			
None	1				
1-9	1.21 (0.59-2.47)				
10-30	0.64 (0.25-1.66)				
**Total fat intake**		0.03			0.02
Normal	1				
Increased	0.65 (0.45 - 0.95)			0.64 (0.43 - 0.94)	
**Socioeconomic level**		0.18			0.15
A1-A2	1			1	
B1-B2	1.20 (0.74-1.94)			1.15 (0.69-1.90)	
C1-C2	0.97 (0.54-1.73)			0.89 (0.50-1.59)	
D-E	1.57 (0.29-8.48)			1.30 (0.27-6.35)	

* Multivariate model adjusted to variables with p<0.25 on bivariate analysis (family history of arterial hypertension, waist circumference, VO_2max_, total fat intake and socioeconomic level).

OR: odds ratio; 95%CI: 95% confidence interval; VO_2max_: maximum oxygen consumption; Q: quartile.

## Discussion

When prehypertension and arterial hypertension were considered together, the prevalences observed in the present study were higher than those reported in an international study by Duncan et al.^32^ (8%) and those reported in the Brazilian studies by Vieira et al.^33^ and Pinto et al.^13^ (11.2% and 4.8%, respectively).

In Brazil, Romanzini et al.^11^ have shown a similar prevalence (18.6%) of high blood pressure, while another study conducted in the city of São Mateus do Sul, Paraná state, has shown a higher high blood pressure prevalence than that in the present study (24.1% for males and 25.6% for females).^20^

Those studies have assessed blood pressure only at one visit. However, as shown in the present study, blood pressure reassessment at a subsequent visit is very important, because blood pressure levels can change from one visit to the other, tending to decrease.

That reduction in blood pressure levels might be due to a decrease in anxiety and familiarization of adolescents with the measuring procedures from the second visit on.^4^ In addition, as approximately 34% of the adolescents in this study had never undergone blood pressure assessment, they could be anxious or even frightened, which could yield increased blood pressure levels. Thus, individuals with high blood pressure levels at the first visit might feel more comfortable with the procedures and measurers from the second visit on.

Regarding the associations, individuals whose both parents were hypertensive had a higher chance to develop high blood pressure. This is in accordance with another study, in which individuals with a history of arterial hypertension had more blood pressure values exceeding the normal limits.^8^ It is worth noting that inherited factors contribute to the development of high blood pressure in adolescents, and, in addition, the adolescent's family setting is shared in regard to behavioral factors,^7,9^ which can contribute to increase blood pressure levels.

Regarding the association of waist circumference and high blood pressure identified in this study, other studies have shown the same tendency.^19,20^ They have evidenced that the 'waist circumference' measure can determine the fat distribution in the abdominal region, being related to metabolic risk, in adults, children and adolescents.^20,34^

Considering the total fat intake, individuals with an increased fat consumption (≥ 30%) have shown a smaller chance to develop high blood pressure, and, thus, total fat intake is a protective factor against high blood pressure.

Contrary to the present study, Guedes et al.^17^ have shown that individuals of both sexes with an inadequate total fat intake had a higher chance to develop high blood pressure. Campos et al.,^18^ however, have reported no significant association of saturated fat and cholesterol intake with arterial hypertension in the city of Curitiba.

Regarding diet composition, the *Dietary Approaches to Stop Hypertension* (DASH), recommended by the *National Institutes of Health* (NIH),^35^ considers other important aspects, such as consumption of whole grains, nuts, fish and poultry. In addition, the NIH emphasizes that the diet should be rich in potassium, magnesium, calcium and fibers, and have a low amount of sodium, red meat, candy, sugar and sugary beverages.^35^

Regarding those characteristics of a healthy diet, it is worth noting that the present study quantified only total fat intake, following the trend of most studies that report separately only a few aspects of the diet. Thus, the result of the present study should be carefully considered, because a high total fat intake can have a high proportion of poly- and monounsaturated fats, which are beneficial to health.^30^ In addition, the individuals analyzed can consume other foods that favor blood pressure reduction, such as whole grains, nuts, fish, poultry, and others rich in potassium, magnesium, calcium and fibers.^35^

The other variables assessed in the present study showed no association with high blood pressure, the literature being controversial about that. Regarding sex, studies have reported higher chances of high blood pressure in males,^10,11^ while others have reported an inverse or nonexistent association.^12,13^

Regarding age, absolute blood pressure levels increase with age in childhood and adolescence, but this seems not to imply increases in high blood pressure levels in that age group.^14^

Considering the daily energy expenditure, the literature has shown no association of physical activity levels with high blood pressure levels.^10,13,16^ Nevertheless, a sedentary lifestyle established during childhood is known to strongly relate to a sedentary lifestyle in adulthood, increasing the susceptibility to the negative repercussions of a sedentary lifestyle in the future, including high blood pressure levels.^3,36^

Regarding cardiorespiratory fitness, Stabelini Neto et al.,^21^ Rodrigues et al.,^37^ and Fernandes et al.^38^ have shown no significant association with high blood pressure in both sexes.

Regarding tobacco use, some studies have reported no association in young individuals of the male sex^16^ and of both sexes,^10^ because a dose-time-response relation is known to exist between tobacco use and the development of related diseases, that is, the risk increases according to the number of cigarettes smoked, especially, regarding the duration of the habit.^39^

Regarding socioeconomic status, the literature is controversial, showing an association of high blood pressure levels with the highest socioeconomic levels,^15^ the lowest socioeconomic levels,^16^ and, even no association.^13^ According to Constanzi et al.,^15^ young individuals of higher socioeconomic levels would eat more and have a more sedentary lifestyle, increasing their chances of developing high blood pressure. Seabra et al.,^40^ however, have reported that individuals of the highest socioeconomic strata tend to practice more physical activities than those less favored.

This study had some limitations, and their analysis can be important for future studies aimed at assessing blood pressure and its determinants. The first concerns the study design, which had a cross-sectional data collection. This type of study can have reverse causality, that is, exposure and outcome are collected simultaneously, making it difficult to determine which preceded the other.

Another limitation concerns the self-reported variables (daily energy expenditure, food intake, smoking, socioeconomic level and family history of arterial hypertension), because they rely on the subjects' understanding about the variables assessed. However, in large-scale studies those variables are extremely useful because they provide the evaluation of several individuals in one single assessment and because they are interesting alternatives when more precise measures cannot be used.

## Conclusion

The present study showed a high prevalence of high blood pressure in adolescents of the city of Curitiba. In addition, it evidenced that taking a second blood pressure measure minimizes the estimates of that outcome among adolescents, probably because of their familiarization with the measuring protocol. Nevertheless, the estimates were greater than those of other Brazilian and international studies.

A positive family history of arterial hypertension and increased waist circumference associated with high blood pressure in adolescents. Therefore, special attention should be paid to young individuals with increased waist circumference, as well as to those with hypertensive parents. Those population subgroups should be the focus of public policies for blood pressure control in the young population, including actions related to the lifestyle adopted in the school and family settings.
